# Dendrimer-Conjugated Glutamate Carboxypeptidase II Inhibitor Restores Microglial Changes in a Rabbit Model of Cerebral Palsy

**DOI:** 10.1159/000530389

**Published:** 2023-03-29

**Authors:** Nirnath Sah, Zhi Zhang, Alicia Chime, Amanda Fowler, Antonio Mendez-Trendler, Anjali Sharma, Rangaramanujam M. Kannan, Barbara Slusher, Sujatha Kannan

**Affiliations:** a Anesthesiology and Critical Care Medicine, Johns Hopkins University School of Medicine, Baltimore, MD, USA; b Department of Natural Sciences, University of Michigan-Dearborn, Dearborn, MI, USA; c Center for Nanomedicine at the Wilmer Eye Institute, Johns Hopkins University School of Medicine, Baltimore, MD, USA; d Department of Chemistry, Washington State University, Pullman, WA, USA; e Johns Hopkins Drug Discovery Program, Johns Hopkins University School of Medicine, Baltimore, MD, USA; f Department of Neurology, Johns Hopkins University School of Medicine, Baltimore, MD, USA

**Keywords:** Dendrimer, Nanoparticle, Glutamate carboxypeptidase II, Microglia, Cerebral palsy

## Abstract

We have previously shown that maternal endotoxin exposure leads to a phenotype of cerebral palsy and pro-inflammatory microglia in the brain in neonatal rabbits. “Activated” microglia overexpress the enzyme glutamate carboxypeptidase II (GCPII) that hydrolyzes N-acetylaspartylglutamate to N-acetylaspartate and glutamate, and we have shown previously that inhibiting microglial GCPII is neuroprotective. Glutamate-induced injury and associated immune signaling can alter microglial responses including microglial process movements for surveillance and phagocytosis. We hypothesize that inhibition of GCPII activity could alter microglial phenotype and normalize microglial process movement/dynamics. Newborn rabbit kits exposed to endotoxin in utero, when treated with dendrimer-conjugated 2-(phosphonomethyl)-pentanedioic acid (D-2PMPA), a potent and selective inhibitor of microglial GCPII, showed profound changes in microglial phenotype within 48 h of treatment. Live imaging of hippocampal microglia in ex vivo brain slice preparations revealed larger cell body and phagocytic cup sizes with less stable microglia processes in CP kits compared to healthy controls. D-2PMPA treatment led to significant reversal of microglial process stability to healthy control levels. Our results emphasize the importance of microglial process dynamics in determining the state of microglial function in the developing brain and demonstrate how GCPII inhibition specifically in microglia can effectively change the microglial process motility to healthy control levels, potentially impacting migration, phagocytosis, and inflammatory functions.

## Introduction

Glutamate is the major excitatory neurotransmitter in the mammalian central nervous system, and increased extracellular glutamate can result in injury and death of neurons. A major source of glutamate in the nervous system is from hydrolysis of the abundant neuropeptide N-acetylaspartylglutamate (NAAG) to N-acetylaspartate and glutamate by the enzyme glutamate carboxypeptidase II (GCPII) [1, 2]. We have previously shown that although normal microglia constitutively express very low levels of GCPII, they are rapidly upregulated in “activated” glia [3, 4]. In the rabbit model of maternal inflammation-induced cerebral palsy (CP) and in a mouse model of neonatal hypoxic-ischemic brain injury, we have shown that GCPII expression increases in “activated” microglia in the neonatal brain, potentially leading to glutamate excitotoxicity and neuroinflammation [3, 4].

Inhibition of GCPII has been shown to be neuroprotective in preclinical models of pathological conditions like traumatic brain injury, stroke, epilepsy, amyotrophic lateral sclerosis, and neuropathic pain [5–11]. Additionally, GCPII inhibition increases extracellular levels of NAAG, decreases the release of glutamate through the effect of NAAG on presynaptic metabotropic glutamate receptor (mGluR) 3, and leads to increased release of TGFβ [12, 13]. NAAG has also been shown to be neuroprotective in neonatal brain injury [14, 15]. Therefore, GCPII inhibition is a potent therapeutic target with translational implications in neonatal brain injury [16, 17]. In animal models of neurologic diseases, selective GCPII inhibitor 2-(phosphonomethyl)-pentanedioic acid (2-PMPA) was shown to enhance brain NAAG levels and improve cognitive function [9, 11, 18]. Despite promising therapeutic data in preclinical models, 2-PMPA exhibits poor pharmacokinetics and brain bioavailability, when injected systemically, making it unsuitable for clinical translation [18]. In this study, we used 2-PMPA conjugated to dendrimer (D-2PMPA) that specifically targets microglial GCPII and has been shown to be more potent and efficacious when compared to the equivalent amount of the free 2-PMPA [18].

Microglia continually protrude and withdraw their processes to perform active surveillance of their microenvironment in physiological conditions and in response to injury [19–21]. These microglia process dynamics are perturbed in neuroinflammation and neurodegeneration in disease-specific ways to modulate microglial functions like phagocytosis or cytokine release [22–24]. Microglia in organotypic slice culture from CP rabbits showed a decrease in migration distance and velocity compared to healthy controls [25]. However, changes in microglial process motility in response to exposure to maternal inflammation and therapy remain unexplored. We hypothesize that exposure to maternal inflammation will lead to alterations in microglial surveillance function as determined by process motility, and targeting GCPII in activated microglia using D-2PMPA would reverse these changes in the neonatal rabbit model of CP.

## Materials and Methods

### Materials

Dendrimer-conjugated 2-PMPA and Dendrimer-2-PMPA-Cy5 was synthesized using generation 4 hydroxyl polyamidoamine (PAMAM) dendrimers (∼4 nm, neutral) followed by purification and characterization as described previously [18].

### Animal Model of CP

New Zealand white rabbits from Robinson Services were bred for the laparotomy surgery on gestational day 28, as described previously [3, 26, 27] and approved by the Animal Care and Use Committee at Johns Hopkins University. Briefly, anesthetized rabbit dams received four intra-uterine LPS injections divided between both horns (0.2 mL volume/horn and total LPS dose ˜ 2,400 EU). On gestational day 30, rabbit dams were induced with intravenous Pitocin (oxytocin, 150 mIU) and kits delivered were labeled and randomly divided between treatment arms. 0.2 mL of either saline or D-2PMPA (20 mg/kg on 2-PMPA basis dissolved in saline) was administered intravenously on day of birth. Following 48 h of IV treatment, the kits were sacrificed for live brain slice preparation or formalin fixation for histological analysis.

### Ex vivo Brain Slice Preparation

Rabbit kits were deeply anesthetized with isoflurane and decapitated. Brains were removed and transferred to oxygenated (95% O_2_/5% CO_2_), ice-cold N-methyl-D-glutamine (NMDG)-based buffer (Buffer 1, in mM: 93 NMDG, 2.5 KCl, 10 MgSO_4_, 0.5 CaCl_2_, 1.2 NaH_2_PO4, 30 NaHCO_3_, 25 glucose, 20 HEPES, 5 sodium ascorbate, 3 sodium pyruvate; PH 7.2–7.4). Coronal brain slices containing the hippocampus (300 μM) were then obtained using a vibratome (VT1200, Leica). Slices were first incubated in the NMDG-based solution at 34°C for 10 min, then transferred and maintained in the same solution for an hour at room temperature before starting subsequent experiment. 50 µL of Tomato lectin, Texas red (Vector Laboratories) was added in 5 mL of Buffer1-containing brain slices and incubated for 30 min while oxygenation. Live imaging is done in solution containing Buffer2 (in mM: 126 NaCl, 3 KCl, 1.5 MgCl_2_, 2.4 CaCl_2_, 1.2 NaH_2_PO_4_, 10 HEPES, 11 glucose) at room temperature. For live imaging, a hippocampal brain slice was transferred to a recording chamber and perfused with oxygenated Buffer2 (2 mL/min). Brain slices were visualized under an upright microscope equipped with laser scanning confocal optics (LSM 880, Zeiss).

### Microglial Process Movement Analysis

Microglial process movement analysis was performed in MATLAB, and the confocal images were processed in Image J as previously described [28]. Z-stacked images were collected from the hippocampus every minute for 10 min, producing 10 time points. Maximum-intensity z-projections were made (20–30 µm thick) and movement artifacts and photobleaching were corrected for before analysis (Fiji). A MATLAB script [28] was used to compare pixels across a series of three images and compares each pixel across consecutive time points to generate a stability index. Any new process extension that remained in the same position for 3 min or more is considered stable, and the stable extensions divided by all extensions is termed as stability index for quantification.

### Live Cell Imaging

Hippocampal or cortical brain slices were imaged at room temperature, at a depth of ∼50 μm in the slice (to avoid studying superficial microglia that had started to become activated by the slicing procedure) using a Zeiss LSM 880 microscope (with a 63X lens). For microglia imaging, brain slices were incubated with lectin for 30 min prior to imaging. Images were 512 × 512 pixels and covered a square field of view 130–150 μm wide. The pixel dwell time was 1 μs. Z-stack of 1–2-μm interval in the vertical direction was used to image 25–35-μm thick brain region of interest. 5–8 rabbit kits per treatment arm were used for live microglia imaging studies.

### Immunohistochemistry

For immune staining of microglia, the rabbits were anesthetized and transcardially perfused with phosphate buffer saline. The brains were collected and post-fixed in 10% formalin overnight and cryoprotected in 30% sucrose solution. Coronal sections (30 µm, 1:6 series) were incubated in 0.3% hydrogen peroxide solution, blocked by 5% normal donkey serum in 0.1 M phosphate buffer saline. Sections were then incubated overnight at 4°C with goat anti-IBA1 (1:500, Abcam, MA, USA), followed by incubation with biotinylated secondary antibodies (1:250; Vector Laboratories, Burlingame, CA, USA). ABC kit and DAB solution from Vector Laboratories (Burlingame) were used according to manufacturer’s protocol to label the microglia. Subsequently, processed sections were dehydrated and cover slipped using mounting medium for imaging. To evaluate the colocalization of D-2PMPA-cy5 in astrocytes or microglia, the sections were incubated overnight at 4°C with chicken anti-GFAP (1:250, Abcam, MA, USA) and goat anti-IBA1 (1:500, Abcam) followed by fluorescent secondary antibodies (1:250; Life Technologies, MA, USA) for 2 h at room temperature. Upon incubation with DAPI (1:1,000, Invitrogen) for 15 min, slides were washed, dried, and cover slipped with mounting medium. Confocal images were acquired with Zeiss ZEN LSM 710 (Zeiss, CA, USA) and processed with ZEN software.

### Statistics

Statistical comparisons were made between groups using Prism software (GraphPad). Sample size was not predetermined but is consistent with previously published studies. Statistical comparisons were done using one-way ANOVA with Tukey’s post hoc comparisons as indicated in the figure legends or unpaired *t* test. Data reported are mean ± SEM.

## Results

### D-2PMPA Localizes in Microglia of CP Kits after Intravenous Administration

**Fig. 1.**
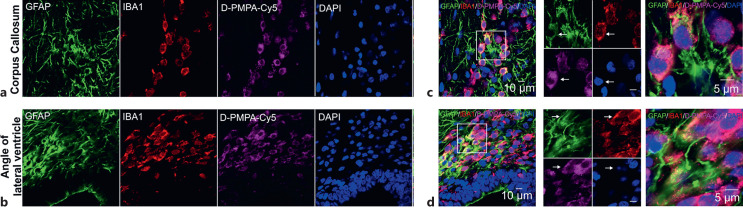
In vivo cellular localization of D-2PMPA-Cy5. CP kits received D-2PMPA-Cy5 injection (i.v.) on PND1 and were sacrificed 24 h post-injection. Coronal brain sections containing D-2PMPA-Cy5 (magenta) were co-stained with GFAP (astrocyte marker, green), IBA1 (microglial marker, red), and DAPI (blue). Colocalization of D-2PMPA-Cy5 in corpus callosum (upper panel, **a**) and angle of lateral ventricle (**b**). **c**, **d** The higher magnification of the region of interest is shown in the right box. Arrows indicate the colocalized GFAP, IBA1, D-2PMPA-Cy5, and DAPI in the higher magnification images.

To evaluate the localization of D-2PMPA, Cy5-conjugated D-2PMPA (20 mg/kg in 0.2 ml) was administered intravenously to CP kits on day of birth (PND1). Kits were sacrificed 24 h post-injection and coronal sections of 30 µm were processed for immunohistochemistry to identify astrocytes (GFAP) and microglia (Iba1). Confocal microscopy showed predominant colocalization of D-PMPA-Cy5 in activated astrocytes and microglia in newborn CP kits (Fig. 1). Angle of lateral ventricle, a highly proliferative zone containing neural progenitor cells (Fig. 1a), and corpus callosum with commissural axon fibers (Fig. 1b) showed morphologically activated microglia containing D-2PMPA-Cy5.

### Enlarged Microglia Cell Body and Phagocytic Cup in CP Kits

**Fig. 2.**
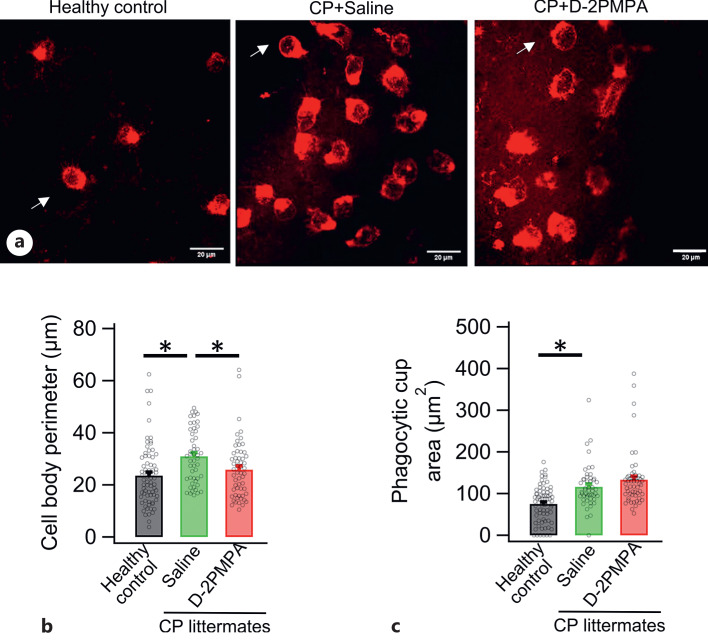
Enlarged microglia cell body and phagocytic cups in CP kits. **a** Confocal images of microglia labeled with lectin in ex vivo live brain slices from healthy control and saline-treated and D-2PMPA-treated CP kits. Cell body perimeter of microglia (**b**) and phagocytic cup area (**c**) is significantly higher in phosphate buffer saline-treated CP kits compared to healthy control. D-2PMPA treatment significantly decreased the microglia cell body perimeter without affecting the phagocytic cup area. *p < 0.05.

To understand the functional effects of D-2PMPA on microglia morphology and process motility, confocal live imaging of microglia in ex vivo brain slice was performed. Morphological features of hippocampal microglia in CP kits after 48 h of treatment with saline or D-2PMPA (20 mg/kg) were compared against the microglia from age-matched healthy control (Fig. 2a). The microglia cell body perimeter in saline treated CP kits is significantly larger (Fig. 2b). Similarly, the phagocytic cup size is more extensive in microglia from CP kits (Fig. 2c). D-2PMPA did not affect the phagocytic cup size but significantly reversed the cell body perimeter within 48 h of treatment (Fig. 2).

### D-2PMPA Stabilizes Microglia Processes in CP Kits

**Fig. 3.**
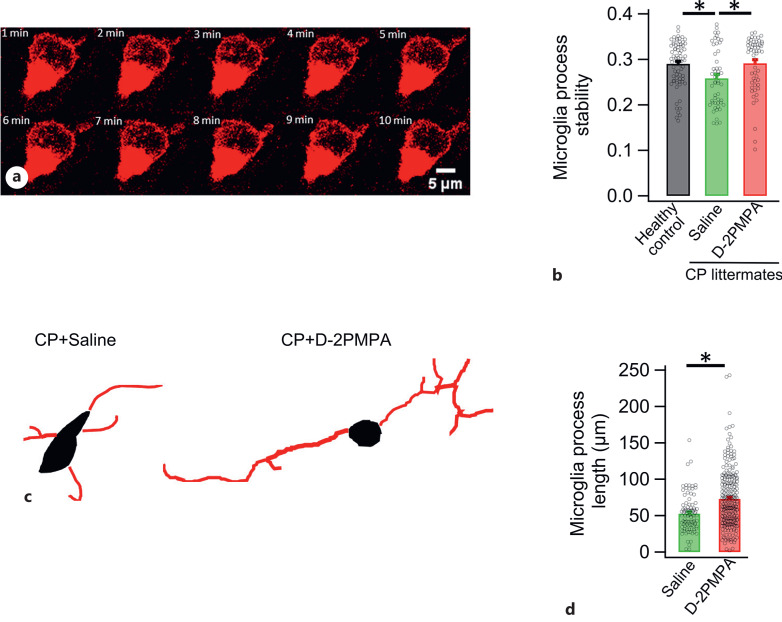
D-2PMPA stabilizes microglia process. **a** Sequential photomicrograph from time-lapse imaging of microglia stained with lectin. Sequential images of this microglia were processed to find the regions where microglia and its processes are changing for different periods of time. **b** CP kits from the same litter were randomly divided into 2 groups and received either saline or D-2PMPA (containing 20 mg/kg 2-PMPA) injection (i.v.) on PND1 and sacrificed 48 h post-injection. D-2PMPA treatment significantly improved microglial stability index to healthy control levels. **c**, **d** Neurolucida tracing of microglia revealed significant increase in microglia process ramification after D-2PMPA treatment. *p < 0.05.

In addition, we characterized microglial process stability over a period of 10 min in live ex vivo brain slices (Fig. 3a–c). Rabbit CP kits were divided into 2 groups and received a single dose of either saline or D-2PMPA (containing 20 mg/kg 2-PMPA) on PND1 and were sacrificed at 48 h posttreatment. Microglia from treatment groups were compared with age-matched healthy controls. Among the saline and D-2PMPA treated groups, there were significant differences in the stability index, a measure of extensions that persisted longer over time (Fig. 3b). We further quantified the microglial processes in formalin-fixed cryo-sections using IBA1 immunohistochemistry. Microglia in the healthy controls and CP kits with and without treatment with D-2PMPA were quantified using neurolucida tracing. Treatment with D-2PMPA significantly increased the microglia process length, implying increased ramification (Fig. 3c).

## Discussion

This study investigates the therapeutic potential of PAMAM dendrimer-conjugated 2-PMPA and its underlying effect on microglial features in the rabbit model of CP. PAMAM dendrimer conjugation with 2-PMPA increased the specificity of targeting 2-PMPA precisely to activated glia, where GCPII is upregulated [3]. This precise microglial targeting of 2-PMPA with D-2PMPA restored microglial process movement and dynamics similar to those of healthy control levels. We conclude that selective inhibition of glial GCPII inhibition by D-2PMPA reinstates microglial process stability that can potentially benefit microglia function and postnatal brain development. This is consistent with our previous studies which demonstrate that inhibiting microglial GCPII using dendrimers is neuroprotective in a mouse model of neonatal hypoxic-ischemic injury [4].

Microglial phagocytosis [29, 30] is increased when there is neurologic injury [31, 32]. A transient increase in phagocytosis and phagocytic cup size is seen in the early postnatal period as part of normal development, playing an important role in synaptic pruning and neurogenesis [33, 34]. As part of microglial maturation, the phagocytic cup size diminishes and processes change from thick to thin [35]. We saw an increase in phagocytic cup size in the endotoxin-exposed kits at 48 h when compared to controls. Although D-2PMPA treatment led to stabilization of the microglial processes and thinner, longer processes, it did not decrease the phagocytic cup size at 48 h after treatment. This may indicate that the increased phagocytic function to clear debris and apoptotic cells after an injury is retained. A temporal evaluation of phagocytic cup size and process length is crucial for a better understanding of how inflammation affects microglial function over time. Pro-inflammatory microglia can exhibit abnormal phagocytosis including engulfing live neurons and neural stem cells, leading to reduced neurogenesis [34, 35]. The decreased microglial process stability seen in our model may also be seen in other injuries such as hypoxic-ischemic encephalopathy where microglia-mediated neuroinflammation may play a significant role in ongoing injury [4, 36].

Selective inhibition of overexpressed GCPII in activated microglia can increase NAAG levels which can exert its neuroprotective effects by activating mGluR3 on microglia and astrocytes [13, 37–39]. mGluR3 is known to regulate cAMP and PKA levels in glia [40, 41]. Such fine regulation of cAMP can control process formation, membrane ruffling, phagocytosis, migration, and chemotaxis of microglia [42–44]. Indeed, mGluR3 activation has been shown to promote Aβ clearance by microglia and astrocytes [45], supporting a significant role of NAAG-mGluR3-cAMP pathway in microglial functions. Similarly, in an in vivo rat model of perinatal brain injury, mGluR3 receptors demonstrated strong regulation of inflammation and selective pharmacological activation of mGluR3 receptors alleviated inflammatory-induced brain injury [46]. Furthermore, mGluR3 activation in astrocytes can be neuroprotective by releasing neurotrophic factors such as TGFβ and BDNF [12, 47]. In an in vitro study using newborn rabbit-mixed glial cultures exposed to LPS, we showed that treatment with D-2PMPA led to an increase in NAAG in the supernatant along with increased cellular expression of mGluR3 and TGFβ [18]. Therefore, increased NAAG can modulate microglial function either directly through its action on microglial mGluR3 or indirectly through the release of TGFβ from astrocytes.

The role and contribution of microglia in neuroinflammation and neuro-excitotoxicity is increasingly appreciated in neurologic injury including CP [30, 48]. Notably, glutamate excitotoxicity can act synergistically with neuroinflammation, resulting in worsening injury in CP [49]. Activated microglia in the CP brain can synthesize and export glutamate substantially [3], leading to abnormal neurotransmission and excitotoxicity. Glutamate produced by activated microglia can bind mGluRs expressed by glia, modulating their function [47]. A thorough understanding of the role of activated microglia and glutamate metabolism will be critical for the future development of novel therapeutics targeting glutamate dysregulation mediated by glia.

This study demonstrates that inhibition of GCPII leads to early changes in microglial function, as indicated by process stability changes. This may have implications for surveillance functions of normal microglia that are crucial for normal development [30, 44]. Future studies will focus on the long-term implications of these changes on microglial surveillance function and migration and sex-related changes in microglial function as a result of injury and therapy.

## Statement of Ethics

The rabbit surgery and treatment protocols used in this study were reviewed and approved by the Johns Hopkins Animal Care and Use Committee, protocol number RB20M322.

## Conflict of Interest Statement

Under license agreements involving Ashvattha Therapeutics, Inc., and the Johns Hopkins University, Drs. Slusher, R. Kannan, S. Kannan and the Johns Hopkins University are entitled to royalty distributions and share ownership related to technology involved in the study discussed in this publication. This arrangement has been reviewed and approved by the Johns Hopkins University in accordance with its conflict of interest policies.

## Funding Sources

This work was funded in part by NIH NINDS Grant No. R01NS093416 (S.K., R.M.K., and B.S.S.).

## Author Contributions

N.S., S.K., B.S., and R.M.K. conceived and designed this study. N.S., Z.Z., A.C., A.F., A.M.-T., and A.S. acquired and analyzed the data presented. N.S., Z.Z., S.K., B.S., and R.M.K. contributed substantially to the manuscript and figure preparation.

## Data Availability

All experimental data are included in this report. Any further information/data will be provided by the corresponding author upon reasonable request.
